# Investigating the impact of media on demand for wildlife: A case study of Harry Potter and the UK trade in owls

**DOI:** 10.1371/journal.pone.0182368

**Published:** 2017-10-04

**Authors:** Diane A. Megias, Sean C. Anderson, Robert J. Smith, Diogo Veríssimo

**Affiliations:** 1 Durrell Institute of Conservation and Ecology, University of Kent, Canterbury, United Kingdom; 2 School of Aquatic and Fishery Sciences, University of Washington, Seattle, Washington, United States of America; 3 Department of Geography and Environmental Engineering, Whiting School of Engineering, Johns Hopkins University, Maryland, United States of America; Universite de Lausanne, SWITZERLAND

## Abstract

In recent decades, a substantial number of popular press articles have described an increase in demand for certain species in the pet trade due to films such as “Finding Nemo”, “Ninja turtles”, and “Harry Potter”. Nevertheless, such assertions are largely supported only by anecdotal evidence. Given the role of the wildlife trade in the spread of pathogens and zoonosis, the introduction of invasive species, the overexploitation of biodiversity, and the neglect of animal welfare, it is crucial to understand what factors drive demand for a species. Here, we investigate the effect the movie industry may have on wildlife trade by examining the relationship between the “Harry Potter” cultural phenomenon and the trade in owls within the United Kingdom (UK). We gathered data from the UK box office, book sales, and newspaper mentions, and examined their relationship with data from three independent sources reflecting the legal ownership of owls in the UK, which is likely to involve several thousands of animals. Additionally, we conducted a questionnaire survey with UK animal sanctuaries to study the presumed mass abandonment of pet owls when the film series ended. Counter to common assertions, we find no evidence that the “Harry Potter” phenomenon increased the legal trade in owls within the UK, even when possible time-lag effects were taken into account. Only one indicator, the number of movie tickets sold, showed a weak but contradictory relationship with demand for owls, with a recorded drop of 13% (95% CI: 3–27%) per 1 SD in tickets sold in the original analysis but an increase of 4% (95% CI: 0–8%) with a one-year lag. In addition, our results suggest that the end of the Harry Potter series did not have a noticeable impact on the number of owls abandoned in UK wildlife sanctuaries, as only two of the 46 animal sanctuaries we contacted independently stated they had seen an increase in owls received and believed this was due to the Harry Potter series. We highlight the importance of further research on the drivers of demand for wildlife to better manage this global trade, and discuss the potential to use films to positively influence behaviour.

## Introduction

The wildlife trade is an expanding global business worth billions of US dollars [1, 2]. Every year, millions of animals and animal products are estimated to be traded to meet consumer demand for food, clothing, decorative items, pets, and traditional medicine [2, 3]. This large scale movement of animals and their parts has been implicated in the spread of pathogens that led to epidemics, the introduction of invasive species, the overexploitation of biodiversity and widespread animal welfare abuses [1, 2]. Most efforts to manage wildlife trade have focused on the supply side, through more robust enforcement and regulation [4, 5]. However, reduced trade barriers and advances in transport and technology, make these measures insufficient [6]. This is why conservationists are increasingly focusing on understanding demand for wildlife as a path to adequately managing the trade.

Demand for wildlife is driven by a number of factors [7–9]. One increasingly discussed issue is the role of movies featuring particular species, as the mainstream media often suggest these impact the wildlife trade by making viewers more interested in purchasing the featured animals [10, 11]. For example, the movie “Finding Nemo” was blamed by the popular press for increasing the trade in clown fish (*Amphiprion spp*.), the species of the film’s main character, despite the movie highlighting marine conservation issues, including overharvesting for the pet trade [11]. Yet, data on the actual trade of clown fish do not support this notion [11].

Several other movies have been implicated in the popular press in the creation of demand for particular species, invariably with limited evidence to support these claims. A recent example arose from reports that the blockbuster Zootopia drove demand for pet fennec foxes in China, which were found to be largely baseless [12]. Therefore, we need to better understand this potential relationship between films featuring animals and the increase in pet trade demand. Here, we focus on the Harry Potter film series and the widely reported story that it led to an increase in the trade of owls in the United Kingdom (UK).

### The Harry Potter phenomenon

The Harry Potter series of books were written by J.K. Rowling and became one of the highest selling book series in the world, with 450 million copies sold [13]. They led to a series of eight films, the first of which was released in 2001, that were watched by millions of people throughout the world [14, 15]. Both the books and films featured several species of owls, which deliver mail to the wizards and act as pets that student wizards bring to school. Harry Potter himself owns a snowy owl (*Bubo scandiacus*). This led to suggestions that the films encouraged British fans to acquire pet owls, a claim repeated by multiple media outlets including the British Broadcasting Corporation and The Huffington Post [16, 17]. Some of the articles further stated that when the film series ended, many owl owners gave up their pet owls to sanctuaries or released them into the wild [18–21]. This allegation was supported by the then Indian Environment Minister, who blamed the Harry Potter books for driving an unsustainable owl trade which lead to a dwindling number of wild owls [22–24]. These accusations prompted J.K. Rowling herself to state “if it is true that anybody has been influenced by my books to think that an owl would be happiest shut in a small cage and kept in a house, I would like to take this opportunity to say as forcefully as I can: please don’t.” [20, 25]. More recently, [26] used undercover surveys in wildlife markets in Indonesia to investigate the demand for pet owls, concluding that the increase in number of owls as a proportion of all birds in the markets suggested a delayed Harry Potter effect. Yet, the fact that exposure or interest to Harry Potter amongst Indonesian consumers was not directly measured, together with the lack of alternative data sources for triangulation made it infeasible to assert a cause-effect relationship.

The UK has a history of owl captive breeding, with large-scale efforts beginning in the 1960s as a response to the decline of wild owl populations, particularly the barn owl (*Tyto alba*) [27]. This effort peaked in the early 1990s, gradually decreasing over the next decade to only a small fraction of its historical size [27, 28]. Currently, the UK continues to have a small active community of owl breeders that commercialize the birds nationally and internationally [29]. Globally, all owl species are listed in the Convention on International Trade in Endangered Species of Wild Fauna and Flora (CITES), the key international treaty regulating the international trade in wildlife. While four species are listed in Appendix I, which lists species already threatened and for which all international commercial trade is prohibited, the remaining are listed in Appendix II, which lists non-threatened species for which trade should be closely monitored [30].

This study aims to assess two hypothesis: (1) the Harry Potter book and film series increased the UK trade in owls, and (2) the end of the Harry Potter film series led to more pet owls being abandoned in the UK. Both of these hypothesis has its genesis in mainstream media reports [16–18]. Assessing the relationship between popular culture and wildlife demand is key to the effective management of wildlife pet demand, and could be a pathway to not only mitigate potential impacts of films but also use them more effectively as tools to positively influence behaviour.

## Methods

We investigated two events: (1) a potential impact of the Harry Potter book and film series in the owl trade within the UK (2) the presumed mass abandonment of pet owls in UK wildlife sanctuaries after the Harry Potter film series ended. We investigated the first issue by looking for a link between indicators of interest in Harry Potter (i.e., sales of cinema tickets and books, mentions in newspapers) and multiple indicators of trade in owls in the UK (i.e. sales of bird rings). We investigated the second issue by surveying British wildlife sanctuaries to measure changes in incidence of owl abandonment and its perceived connection with the Harry Potter phenomenon.

### Data collection

#### Harry Potter interest

To measure the interest in Harry Potter films within the UK, we used a weekly summary of weekend box office grosses available from the British Film Institute website [15]. These figures cover box office grosses from Friday to Sunday. We then calculated monthly total gross incomes for each Harry Potter film and assessed the number of admissions per year by dividing the total gross incomes by the average price of a ticket [31]. We also used data on the yearly UK sales of all Harry Potter books, across all language and versions, obtained from Nielsen Book Services. Lastly we used the Lexis Nexis database, to gather data on the yearly number of mentions in major UK newspapers. We included all available newspapers with at least one million copies sold annually (including Sunday supplements), resulting in the inclusion of The Daily Mail, The Mirror and The Times.

#### The owl trade

We assessed the magnitude of the owl trade using three independent sources: CITES and two key UK suppliers of bird rings—the Independent Bird Register and the British Bird Council. The ringing of Annex A birds, which include owls, is a legal requirement in the UK if the birds are to be sold or used for commercial purposes [32]. We collected monthly data from the Independent Bird Register concerning the number of rings issued by the organisation for snowy owls within the UK from 1994 to 2012. The focus on the snowy owl is due to the species being the most prominent owl specie in the Harry Potter storyline. Similar monthly data were obtained from the British Bird Council for the period 1996–2012 concerning two relevant ring sizes: size “Z” which covers the snowy owl and European eagle owl (*Bubo bubo*), and size “U” which is recommended for barn owl, short-eared owl (*Asio flammeus*) and tawny owl (*Strix aluco*), as well as marsh harrier (*Circus aeruginosus*), carrion crow (*Corvus corone*), female hen harrier (*Circus cyaneus*), hooded crow (*Corvus cornix*) and rook (*Corvus frugilegus*). The data extracted from the CITES online trade database were annual records on the legal trade of the snowy owl and of all owl genera listed by the convention for which a record of a trade associated with the UK was available. This dataset covered the period 1990–2011.

#### The abandonment of owls

To assess the abandonment of pet owls described by the press as a consequence of the termination of the film series, we contacted 117 wildlife sanctuaries between September 2012 and February 2013 to conduct a questionnaire survey. We identified sanctuaries that might have received unwanted owls through Google searches using the keywords “owl”, “bird of prey”, and “wildlife”, with “sanctuary”, “rescue centre”, and “care centre” (see S1 Table).

### Data analysis

We modelled three measures of demand for owls in the United Kingdom as a function of the number of Harry Potter books sold, Harry Potter box office movie tickets sold, and mentions of Harry Potter in UK newspapers. We chose to model the effect of books, movies, and newspapers separately because of the different time periods of data available and the limited number of data points. We fitted Generalized Additive Models (GAMs) of the form:

$$\text{log}\left(E\left({\text{Owl}}_{i}\right)\right)={\text{Saliency}}_{\text{i}}+f\left({\text{Year}}_{i}\right),\;{\text{Owl}}_{i}∼\text{Quasipoisson},$$

where Saliency_*i*_ represents either the number of books purchased, movie tickets purchased, or newspaper mentions in year *i* and $E\left({\text{Owl}}_{\text{i}}\right)$ represents the expected value of the owl response variable in year *i*. We modeled time (Year_*i*_) as a smooth spline function, represented by *f*, using the package mgcv [33] for the statistical software R [34]. We limited the number of knots in the smooth function to three due to the limited number of data points. This smooth function controlled for other nonlinear effects through time beyond the included covariates. Modelling the effect of year was sufficient to remove any substantial autocorrelation in the residuals. We initially fitted our models with a Poisson error distribution but found that the data were overdispersed. Therefore, we fitted our final models with a Quasipoisson error distribution. The data from CITES were not quantitatively evaluated, since the small (eight) number of non-zero data points meant the models would be statistically underpowered.

## Results

### Owls purchased

**Fig 1:**
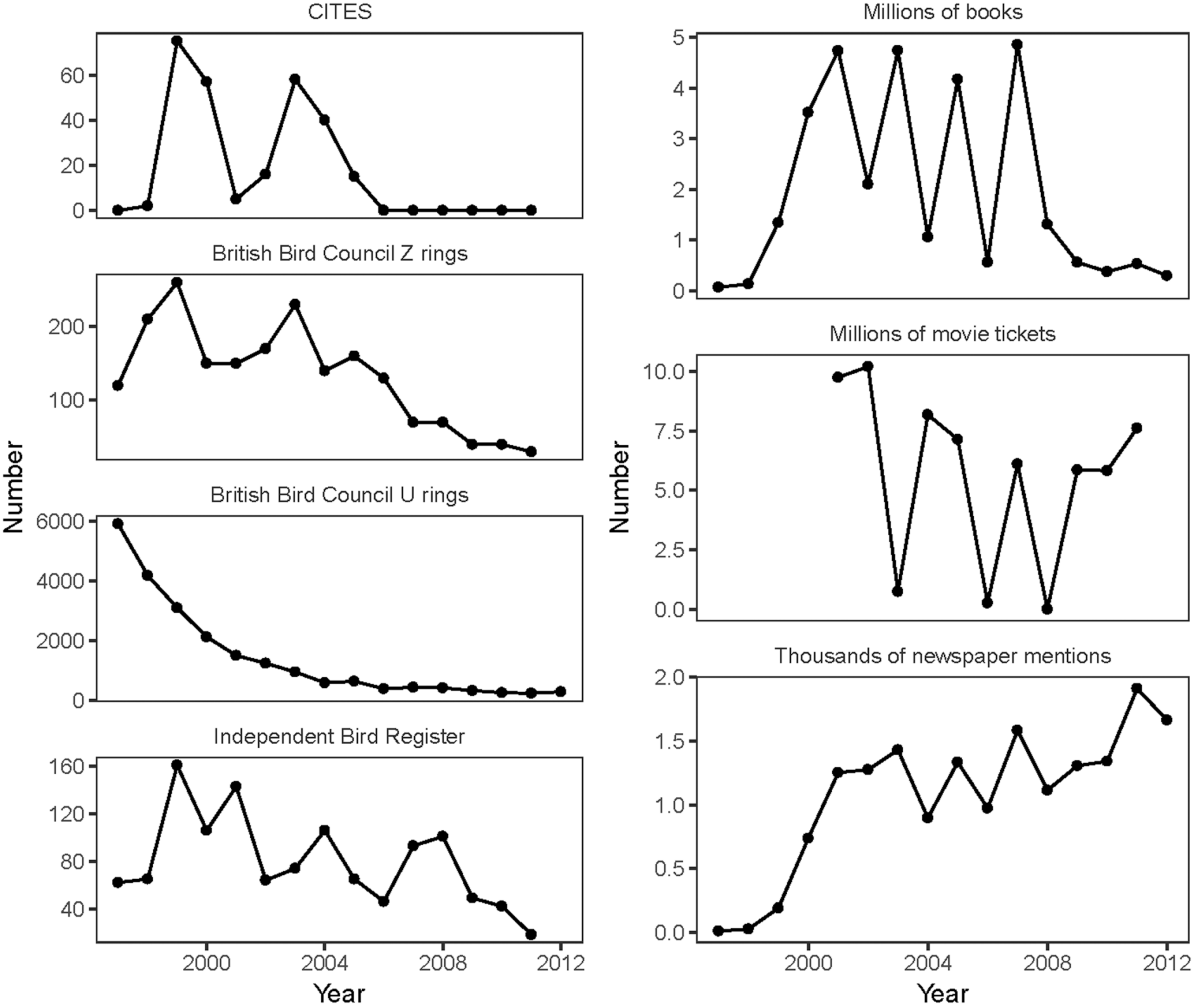
Indicators of owl demand and Harry Potter saliency in the UK. Clockwise: Yearly number of Harry Potter books sold in the UK across all versions and languages; Yearly number of tickets sold for Harry Potter movies in the UK; Yearly number of newspaper mentions in main UK newspapers; Yearly number of bird rings for snowy owls sold by the Independent Bird Register; Yearly number of Z bird rings for snowy owl and European Eagle owl sold by the British Bird Council; and yearly number of U bird rings for Barn owl, short-eared owl, Tawny owl, Marsh harrier, Hen harrier (female), Carrion crow, Hooded crow and rook sold by the British Bird Council.

**Fig 2:**
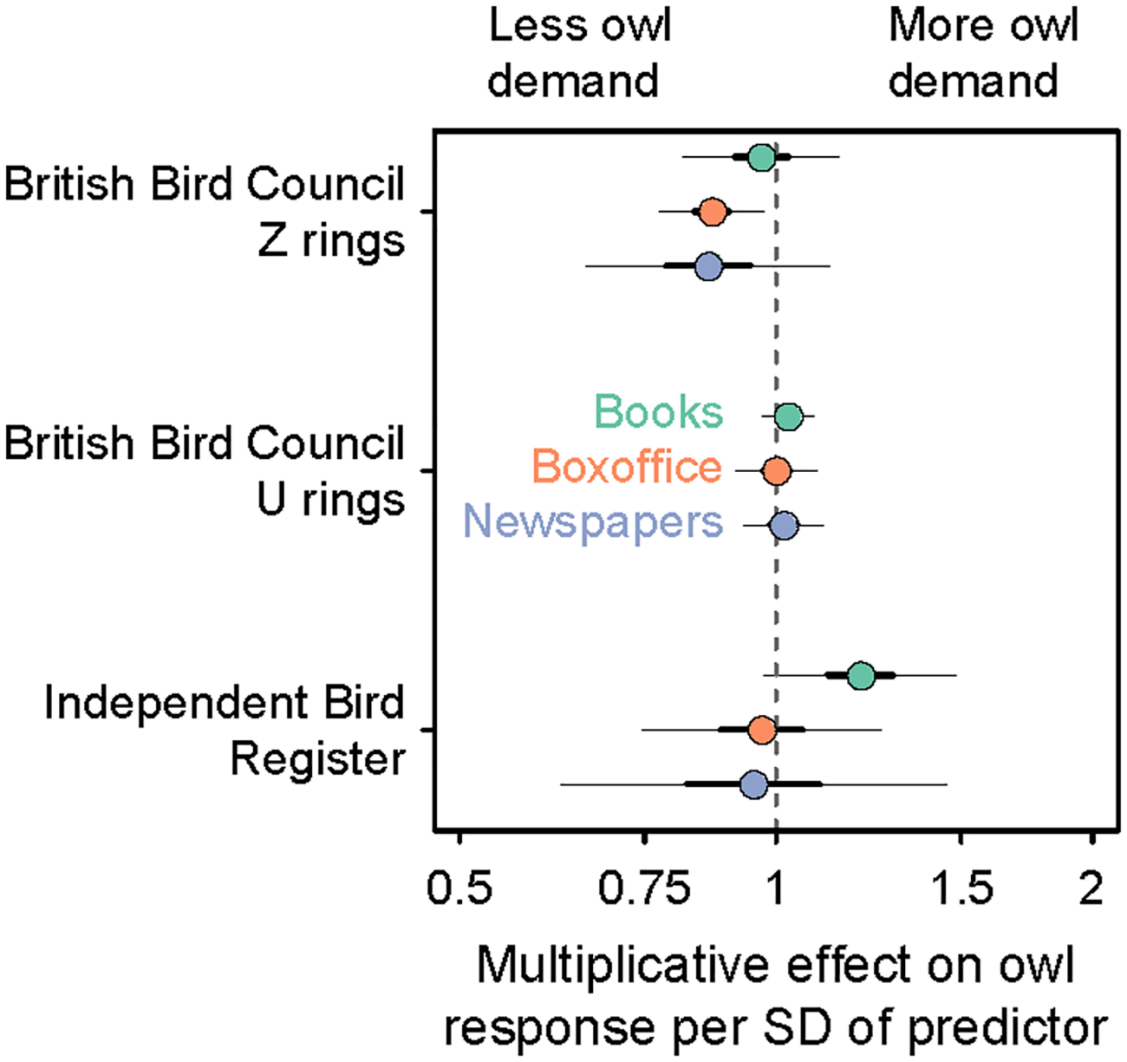
Relationship between Harry Potter popularity and owl demand. Relationship between three measures of Harry Potter popularity (yearly number of Harry Potter books sold in the UK across all versions and languages; yearly number of tickets sold for Harry Potter movies in the UK, and yearly mentions in UK newspapers) and three measures of owl demand (yearly number of bird rings for snowy owls sold by the Independent Bird Register; yearly number of Z bird rings for snowy owl and European Eagle owl sold by the British Bird Council; and yearly number of U bird rings for barn owl, short-eared owl, tawny owl, marsh harrier, hen harrier (female), carrion crow, hooded crow and rook sold by the British Bird Council). Dots represent the multiplicative effect of an increase in one standard deviation of a predictor variable on the response variables. For example, a value of 1.2 would represent an expected 20% increase in owl demand per one standard deviation increase in a predictor. Thick and thin line segments represent 50% and 95% confidence intervals. These coefficients are derived from a model that also includes a nonlinear smoother for time.

**Fig 3:**
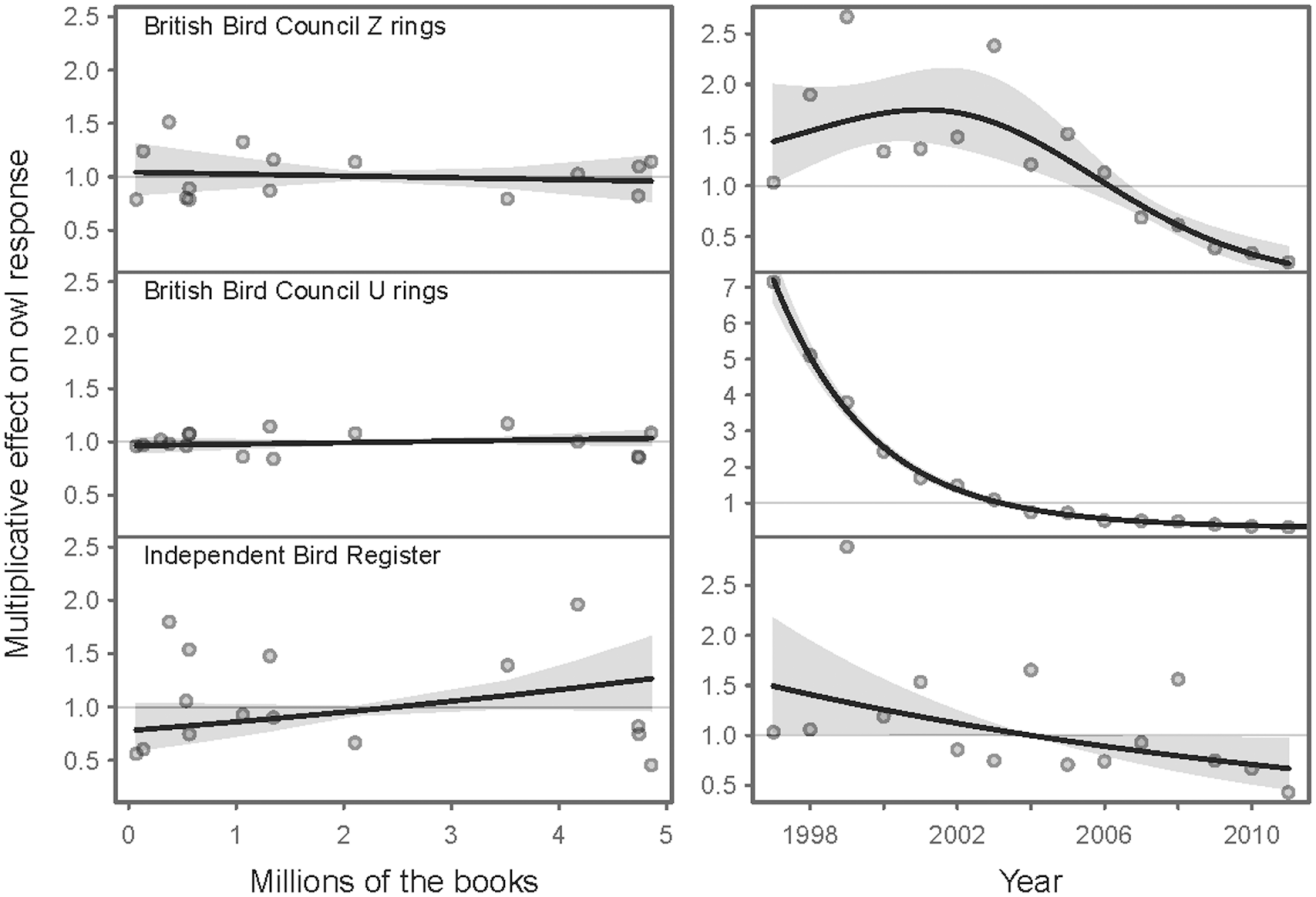
Effect of the number of Harry Potter books purchased on three measures of demand for owls in the United Kingdom. Shown are the individual effects (lines) and +/- two standard errors (shading). Partial residuals are shown with dots. The smooth term controlling for time is shown in the right column. The effects shown are multiplicative and the predictors were centred before model fitting by subtracting their means. Therefore, a value of 1.2, for example, would indicate a 20% increase in demand compared to the effect at the mean of the predictor.

The number of owls sold decreased through time for all demand indicators considered (Fig 1). Indicators of the popularity of Harry Potter varied widely through time, with the exception of newspaper coverage, which generally increased over time (Fig 1). None of the popularity indicators of Harry Potter were a strong predictor of owl demand (Fig 2), even if we lagged the saliency data (books, tickets, or newspaper mentions) forward one year to account for any delays in response to variations in popularity (S1 Fig). The only potential exception was the number of movie tickets sold, which in the initial analysis was weakly linked with less (13%, 95% CI: 3–27% per 1 SD in tickets sold) demand for owls, as measured by the number of Z rings sold by the BBC, and in the time-lagged analysis was weakly linked with more (4%, 95% CI: 0–8% per 1 SD in tickets sold) demand for owls according to the same demand indicator. Across all three predictors, the smooth term controlling for time was a much stronger predictor of the decrease in demand for owls than any of the three Harry Potter popularity indicators (Fig 3, S2 and S3 Figs).

### Abandonment of pet owls

**Fig 4:**
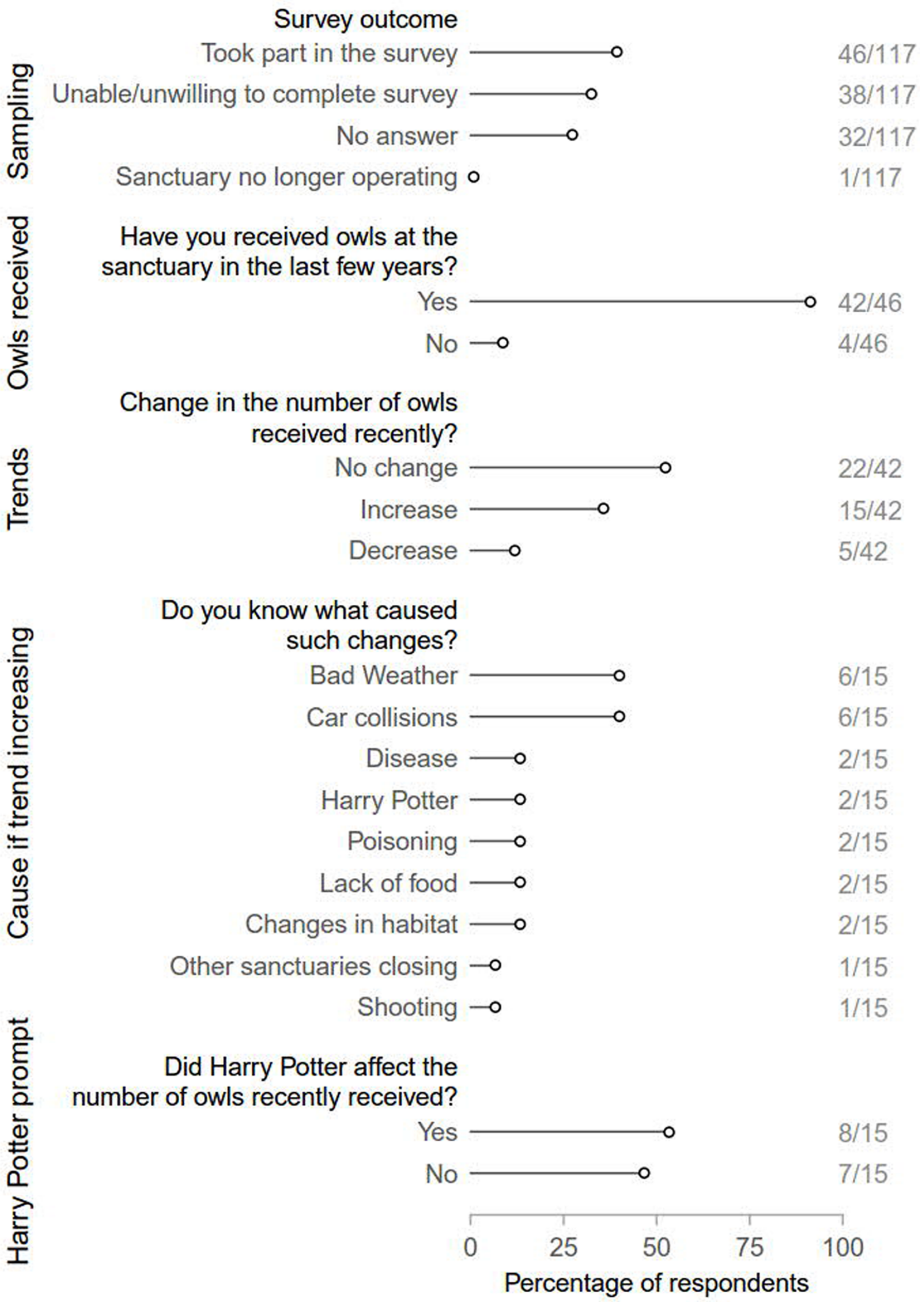
Results of survey on owl abandonment. Numbers at the right indicate response counts and sample sizes for each answer.

We attempted to contact 117 wildlife sanctuaries and were able to reach 85. Of the sanctuaries we were able to reach, one was no longer operating, 19 refused to take part in the survey and a further 19 did not complete the survey, leaving a total of 46 complete responses. Of these, 42 stated they had received owls in the recent past, with a third noting an increase in the number of owls received (Fig 4). The main causes for this increase were identified as poor weather conditions and car collisions (Fig 4). Only two respondents mentioned, without being prompted, Harry Potter films as a cause for the increase in received owls. However, after being prompted, about half (8/15) of those that mentioned an increase in the number of owls received, said they believed the Harry Potter phenomenon had played a role.

## Discussion

We aimed to understand the role played by the Harry Potter cultural phenomenon in increasing the number of owls bought as pets in the UK, and later on, in generating a wave of abandonments of owls in wildlife sanctuaries. Contrary to what was widely reported in the popular press, we found no evidence that the Harry Potter films had a substantial impact on either of these aspects within the United Kingdom.

### Owls purchased

Our results suggest that Harry Potter popularity is a poor predictor of demand for owls, even if the analysis takes into account a potential time-lag between exposure to the Harry Potter cultural phenomenon and the purchase of the owl. Yet, the strong relationship between time and owl demand suggests that other causal mechanisms are driving the trade in owls. One alternative explanation relates to the decrease in interest in the breeding of barn owls in the UK. From the estimated 3000 birds released annually in 1992 that triggered the need for a licensing scheme, this practice progressively lost popularity, with the licensing scheme ending in 2002 due to lack of participation [27, 28]. This trend coincides with the 80% reduction in bird rings sold for barn owls documented by the British Trust for Ornithology between 1993 and 2000 [35], which suggests that the birds were not only not being released but were also not being bred. Lastly, the inclusion in our models of a variable measuring the saliency of the Harry Potter series in the press also lets us test the hypothesis raised by [12] that it was this factor, and not the movies themselves, that was driving public interest in particular species. Our results do not support this hypothesis, suggesting instead that, in this case, press saliency did not translate into actual trade.

The fact that this study is based only on data from the legal trade may mean it only covers a portion of the owl trade. Indeed, a few sources suggesting an increase in demand for pet owls following the Harry Potter series in India and Indonesia highlight the illegal nature of the owl trade in these countries [22, 23, 26]. However, in the UK the breeding of owls is not only legal but commonly practiced [27]. Thus we would expect the legal market to absorb the majority of any increase in demand. This openness also facilitates the testing of our hypothesis, as changes are more likely to be documented, particularly in a developed country such as the UK, where there is comparatively stronger environmental governance.

### Abandonment of pet owls

Concerning the survey to assess the abandonment of owls, only 46 wildlife sanctuaries out of the 85 contacted, took part in the survey. This reduced proportion of respondents may have impacted the representativeness of our sample, potentially skewing our sample towards larger and better staffed facilities, where staff were able to take the time to complete the survey. On the other hand, we would expect those facilities to receive the majority of animals, thus bringing our estimates closer to the national total than would be expected from the proportion of centres sampled. Another aspect to take into account is that not all UK sanctuaries are part of the list used in this research, which means our work focuses only on a sample of these institutions. Future research should take into account also the lists of animal rescue centres compiled by institutions such as the British Wildlife Rehabilitation Council, Royal Society for the Prevention of Cruelty to Animals and Help Wildlife.

Considering the small proportion of respondents that independently linked the Harry Potter phenomenon with owl abandonment, the survey results suggest that the end of the Harry Potter series did not play a substantial role in increasing the number of owls abandoned in sanctuaries. Despite this overall picture, it is interesting that after being prompted, six respondents did express a conviction that the Harry Potter phenomenon had a major impact on owl species abandonment. For example, one respondent stated that “as for any other film featuring animals, people want them as pets and then get rid of them when they realize they don’t actually make such good pets”. While it is possible that the prompt reminded respondents of less salient experiences where owl abandonment was indeed linked to Harry Potter, it is also possible for these answers to be influenced by a type of priming effect where exposure to the media coverage makes the respondent more likely to make that same causal association [36]. In either case we can conclude that at most the impact of the Harry Potter films was very limited.

Even if we accept that the Harry Potter films were responsible for some abandonment of owls in a few sanctuaries, our results suggest it is not reasonable to generalize this trend to the whole UK. Even after prompting, the sanctuaries that received an increasing number of owls and believed that Harry Potter films were partly responsible only represented about one sixth of all participants. Furthermore, based on the causes mentioned for owls to be received at wildlife sanctuaries, most owls seem to be wild animals rather than pets, confirming the lack of a link between the films and the trade in owls.

### Questioning the hype

Our results raise questions as to why this issue was so widely reported by the popular media. An analysis of the evidence mentioned in those articles reveals that most relied on anecdotal evidence from interviews of a small number of individuals, whose names appear repeatedly in multiple articles. Similarly, the evidence presented around the rising number of owls abandoned in sanctuaries after the film series ended relies on information from only four sanctuaries, none of which we were able to contact. Thus, the extrapolation of results obtained from such a thin evidence base to a national level is questionable, and might explain the difference between what articles describe and what the present study shows. This speculation is however not without consequence, and the presumed impact of films such as “Finding Nemo” has, for example, led to the launch of campaigns [37, 38] and press releases by several international organizations on the hypothetical impacts of its recent sequel “Finding Dory”, launched in June 2016.

A broader evidence base is needed to better understand the influence that different types of media can have on the demand for natural resources, and allow for a more data-driven narrative to emerge around this issue. This is, however, made difficult by the lack of access to high quality datasets and the lack of standardized reporting on commercial transactions involving wildlife. For example, CITES does not record transactions within the European Union and therefore only specimens imported from outside the EU are included in the figures collected. In another example, the data for size U rings obtained from the British Bird Council covered three owl species; however, this ring size is also recommended for other bird species, which may have introduced noise in our analysis. Given these shortcomings, it is crucial to use multiple datasets collected by independent sources to triangulate the results obtained. It is this use of multiple datasets to measure both exposure to the Harry Potter and owl trade that sets this research apart from prior studies [see 26] and provides the most robust evidence to date of a lack of impact of the Harry Potter phenomenon on the trade of owls.

### Tackling trade from the demand side

We believe that the film industry could play an important role in influencing behaviour, through, for example, the shaping of social norms, as they have the ability to reach large audiences [10]. To this point, it is worth noting that the news coverage linking the Harry Potter phenomenon to the owl trade surfaced only after the release of the first Harry Potter movie, despite it being preceded by the bestselling book series in history. This highlights the power that films have in comparison to other information channels. It is also worth reflecting on the geographic specificity of the narrative linking Harry Potter to the owl trade, as it was mostly present in the UK, where J.K Rowling is from and where the series had the most popularity. The exception to this was the declaration of the Indian Ministry of Environment, which was unique also in its emphasis on the link between Harry Potter and the illegal wildlife trade. This suggests that even in highly globalised cultural phenomena the outcomes are likely to differ by region, depending on the cultural context.

The complexities of dealing with demand for trade in wildlife will no doubt require collaborations between film maker’s and conservationists, especially those working within the social sciences areas such as marketing, economics, anthropology, and psychology [39]. Such efforts should work to ensure that future films not only do not have a negative impact on wildlife, but act as tools to shape social norms around pet ownership. In this way, future movies can become powerful allies in mitigating the impacts on wildlife trade on species conservation, animal welfare and public health.

## Supporting information

S1 Fig Relationship between Harry Potter popularity and owl demand with a one-year time lag. Relationship between three measures of Harry Potter popularity (yearly number of Harry Potter books sold in the UK across all versions and languages; yearly number of tickets sold for Harry Potter movies in the UK, and yearly mentions in UK newspapers) and three measures of owl demand (yearly number of bird rings for snowy owls sold by the Independent Bird Register; yearly number of Z bird rings for snowy owl and European Eagle owl sold by the British Bird Council; and yearly number of U bird rings for barn owl, short-eared owl, tawny owl, marsh harrier, hen harrier (female), carrion crow, hooded crow and rook sold by the British Bird Council). Dots represent the multiplicative effect of an increase in one standard deviation of a predictor variable on the response variables. For example, a value of 1.2 would represent an expected 20% increase in owl demand per one standard deviation increase in a predictor. Thick and thin line segments represent 50% and 95% confidence intervals. These coefficients are derived from a model that also includes a nonlinear smoother for time.
(TIF)

S2 Fig Effect of the number of Harry Potter movie tickets purchased on three measures of demand for owls in the United Kingdom. Shown are the individual effects (lines) and +/- two standard errors (shading). Partial residuals are shown with dots. The smooth term controlling for time is shown in the right column. The effects shown are multiplicative and the predictors were centred before model fitting. Therefore, a value of 1.2, for example, would indicate a 20% increase in demand compared to the effect at the mean of the predictor.
(TIF)

S3 Fig Effect of the number of Harry Potter newspaper mentions on three measures of demand for owls in the United Kingdom. Shown are the individual effects (lines) and +/- two standard errors (shading). Partial residuals are shown with dots. The smooth term controlling for time is shown in the right column. The effects shown are multiplicative and the predictors were centred before model fitting. Therefore, a value of 1.2, for example, would indicate a 20% increase in demand compared to the effect at the mean of the predictor.
(TIF)

S1 Table List of wildlife sanctuaries contacted to assess the abandonment of pet owls described in the UK press as a consequence of the end of the Harry Potter film series. (DOCX)

S1 File Raw data underlying this study. (DOCX)
