# Supplementary figures and images for: Investigating the impact of media on demand for wildlife: A case study of Harry Potter and the UK trade in owls
Source: PLoS One. 2017 Oct 4;12(10):e0182368. doi: 10.1371/journal.pone.0182368 (PMC5627891; doi:10.1371/journal.pone.0182368)

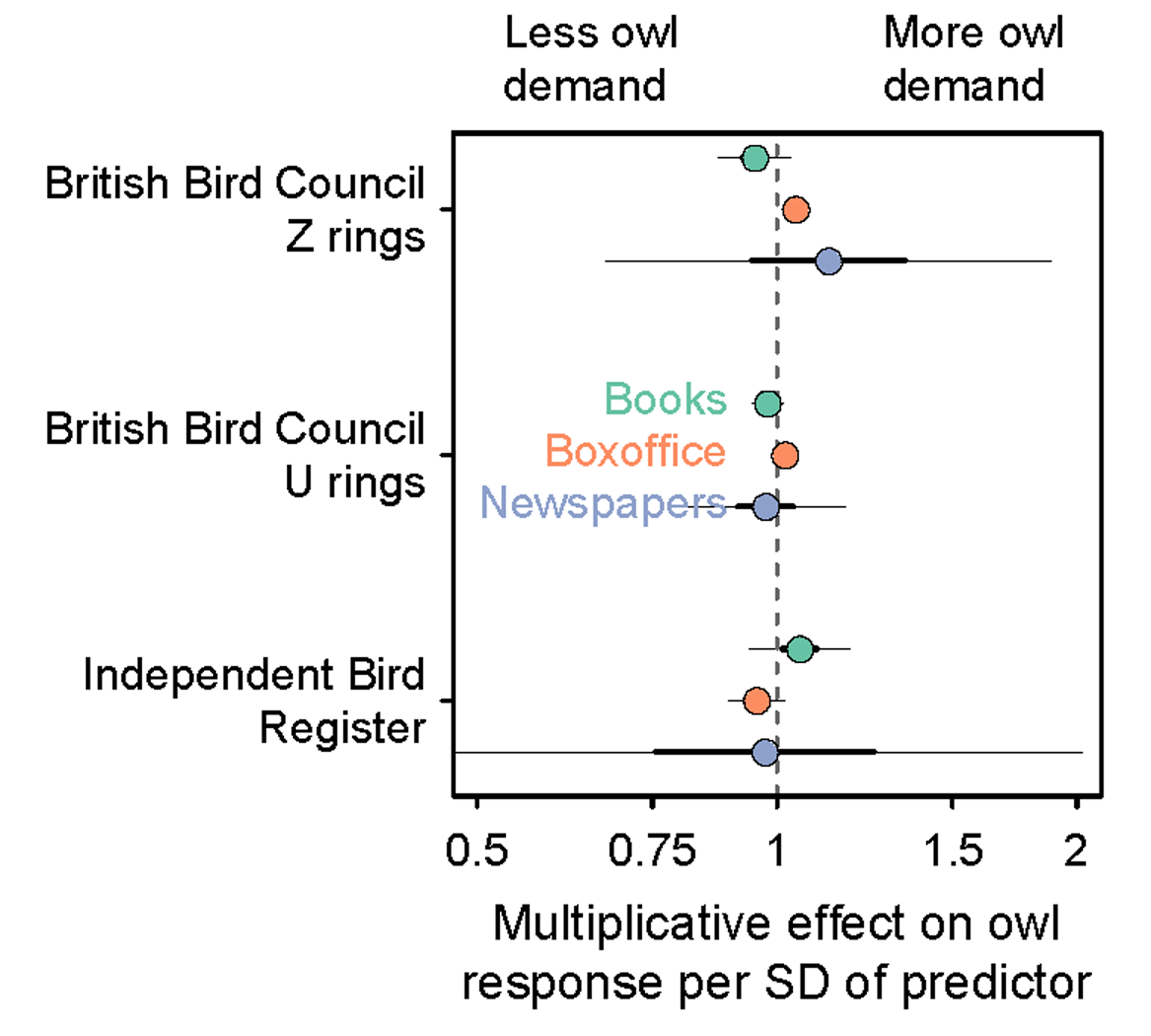

Supplement: S1 Fig — Relationship between three measures of Harry Potter popularity (yearly number of Harry Potter books sold in the UK across all versions and languages; yearly number of tickets sold for Harry Potter movies in the UK, and yearly mentions in UK newspapers) and three measures of owl demand (yearly number of bird rings for snowy owls sold by the Independent Bird Register; yearly number of Z bird rings for snowy owl and European Eagle owl sold by the British Bird Council; and yearly number of U bird rings for barn owl, short-eared owl, tawny owl, marsh harrier, hen harrier (female), carrion crow, hooded crow and rook sold by the British Bird Council). Dots represent the multiplicative effect of an increase in one standard deviation of a predictor variable on the response variables. For example, a value of 1.2 would represent an expected 20% increase in owl demand per one standard deviation increase in a predictor. Thick and thin line segments represent 50% and 95% confidence intervals. These coefficients are derived from a model that also includes a nonlinear smoother for time. (TIF) [file pone.0182368.s001.tif]

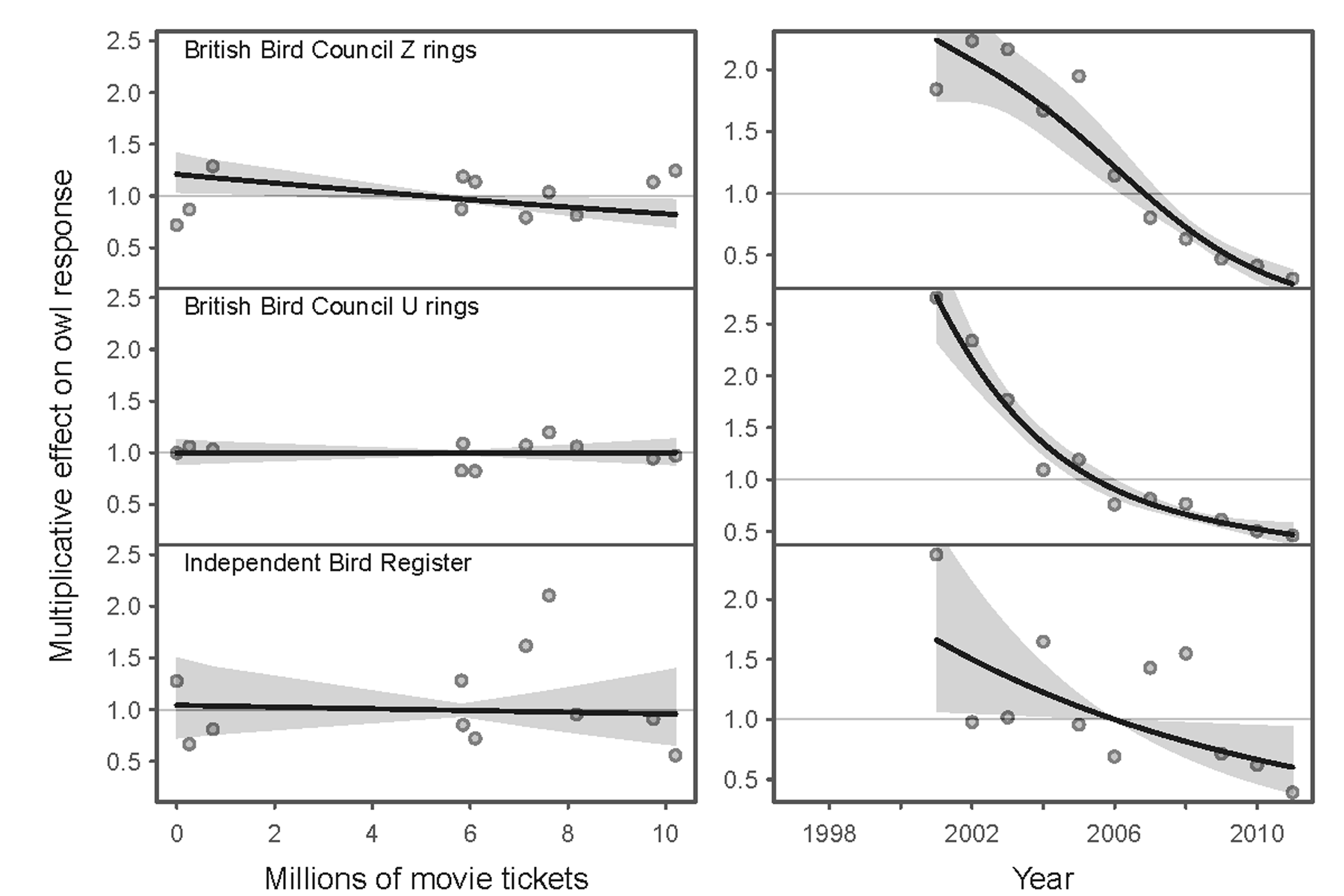

Supplement: S2 Fig — Shown are the individual effects (lines) and +/- two standard errors (shading). Partial residuals are shown with dots. The smooth term controlling for time is shown in the right column. The effects shown are multiplicative and the predictors were centred before model fitting. Therefore, a value of 1.2, for example, would indicate a 20% increase in demand compared to the effect at the mean of the predictor. (TIF) [file pone.0182368.s002.tif]

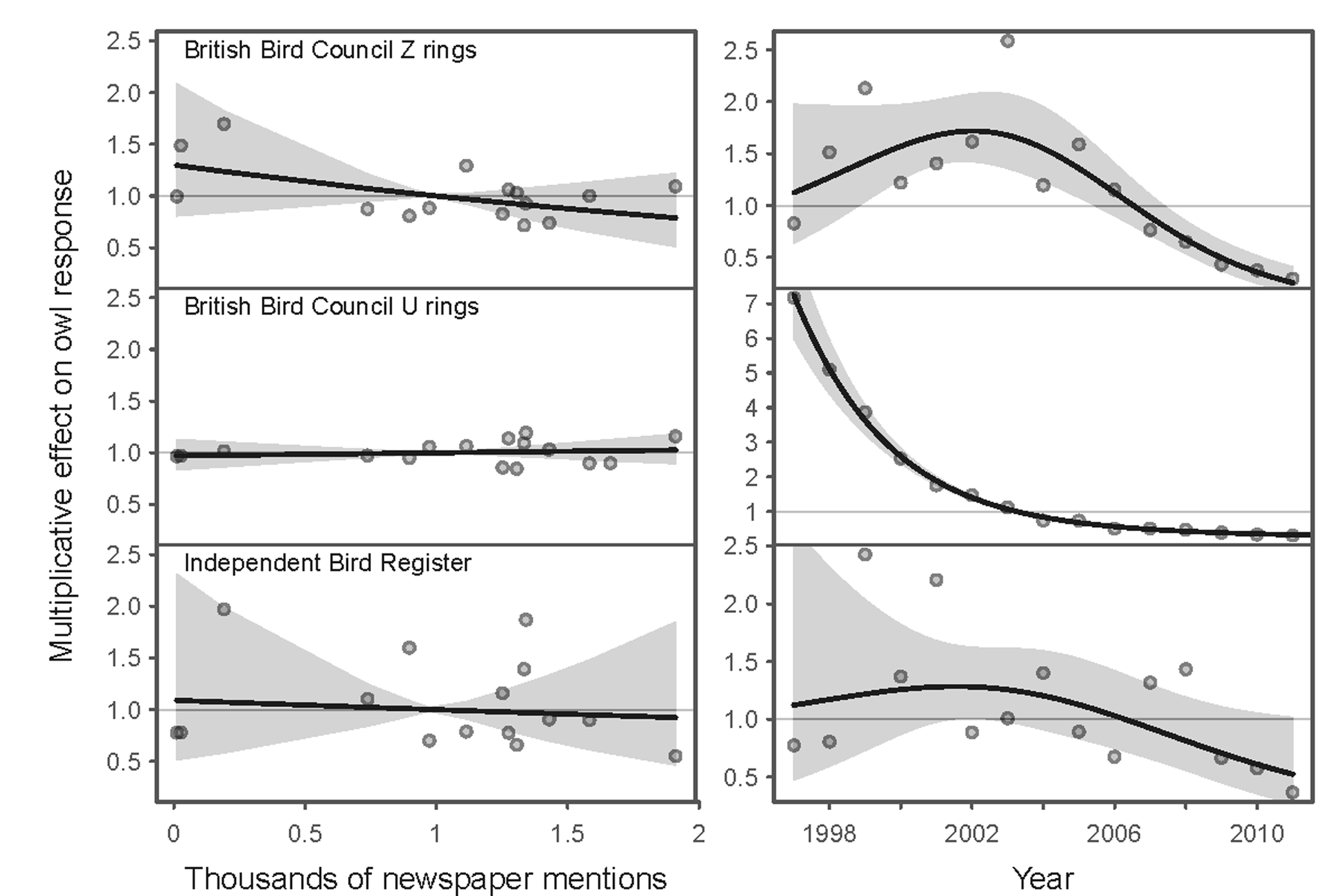

Supplement: S3 Fig — Shown are the individual effects (lines) and +/- two standard errors (shading). Partial residuals are shown with dots. The smooth term controlling for time is shown in the right column. The effects shown are multiplicative and the predictors were centred before model fitting. Therefore, a value of 1.2, for example, would indicate a 20% increase in demand compared to the effect at the mean of the predictor. (TIF) [file pone.0182368.s003.tif]
