# Supplementary material for: Investigating the impact of media on demand for wildlife: A case study of Harry Potter and the UK trade in owls
Source: PLoS One. 2017 Oct 4;12(10):e0182368. doi: 10.1371/journal.pone.0182368 (PMC5627891; doi:10.1371/journal.pone.0182368)
Supplement: S1 Table — (DOCX) [file pone.0182368.s004.docx]

| **Sanctuary name** | **Location of the sanctuary (county)** | **Respondent to our survey** | **Contact information of sanctuaries which responded to our survey** |
| --- | --- | --- | --- |
| Animals in need | Northamptonshire | No |  |
| Ark on the edge | Durham | Yes | 00441833630505 |
| Arundel Wildlife Rescue | Sussex | No |  |
| Ashley Health animal centre | Dorset | No |  |
| Barn Owl bill | Lancashire | No |  |
| Bedfordshire Wildlife Rescue | Bedfordshire | Yes | 00441582527465 |
| Bedhampton Bird and Wildlife rescue centre | Hampshire | No |  |
| Birdie mama's Sanctuary | Buckinghamshire | No |  |
| Block Fen animal centre | Cambridgeshire | No |  |
| Blyth Wildlife Rescue | Northumberland | Yes | 00447910643122 |
| Brent lodge Bird & Wildlife Trust | Sussex | Yes | 00441243641672 |
| British Trust for Ornithology | Norfolk | No |  |
| British Wildlife Rescue Centre | Staffordshire | Yes | 00441889271308 |
| Bryn-y-Maen animal centre | Conwy (Wales) | No |  |
| Burton Wildlife Rescue | Staffordshire | Yes | 00447780742748 |
| Cedarwood Wildlife Care | Hampshire | No |  |
| Cheam Wildlife Care | Surrey | No |  |
| Chrissie's Owls | Oxfordshire | Yes | 00441993891618 |
| Cotswold Owl Rescues Trust | Gloucestershire | No |  |
| Cuan House Wildlife rescue centre | Shropshire | No |  |
| Cullompton Wildlife Centre | Devon | Yes | 00441884839265 |
| Denbighshire Avian Rescue | Denbighshire (Wales) | No |  |
| Dorset Wildlife Rescue | Dorset | Yes | 00447502299362 |
| Eagle Heights | Kent | No |  |
| East Sussex Wildlife Rescue and ambulance service | Sussex | No |  |
| East Winch wildlife centre | Norfolk | No |  |
| Elstead bird Rescue Centre | Surrey | No |  |
| Endangered Species of Wildlife Animals | Hampshire | No |  |
| Fallhead Falconary | Yorkshire | No |  |
| Folly Wildlife Rescue Tunbridge Wells | Kent | Yes | 00441892543213 |
| Foxy Lodge Wildlife Rescue | Norfolk | Yes | 00441493384237 |
| Fur & Feather Wildlife Trust | Kent | Yes | 00447906315365 |
| Gentleshaw Wildlife Centre | Staffordshire | No |  |
| Glebe Wildlife Centre | Yorkshire | No |  |
| Great Ayton animal centre | Yorkshire | No |  |
| Great Manchester animal Hospital | Greater Manchester | No |  |
| Greenman Wildlife Care | Hampshire | No |  |
| Harmsworth memorial animal hospital | London | No |  |
| Harper Asprey Wildlife Rescue | Surrey | No |  |
| Hart wildlife Rescue | Hampshire | No |  |
| Hartland Wildlife rescue | Devon | No |  |
| Headcorn Wildlife Rescue | Kent | Yes | Sanctuary is no longer operating – no contact information could be retrieved |
| Hereford Owl Rescue | Herefordshire | No |  |
| Holderness Raptor Rescue | Yorkshire | Yes | 00441482838096 |
| Kays Hill Animal Sanctuary | Durham | Yes | 00441388834996 |
| Kirkleatham Owl Centre | Yorkshire | No |  |
| Leicestershire Wildlife Hospital Trust | Leicestershire | Yes | 00447951285366 |
| Leybourne Animal Centre | Kent | Yes | 00443001230751 |
| Little Foxes Wildlife rescue | Oxfordshire | No |  |
| London WildCare | Greater London | Yes | 00442086476230 |
| Mallydams wood | Sussex | No |  |
| Meripac Ltd Animal & Bird Housing | Middlesex | No |  |
| Moonpenny Farm | Yorkshire | Yes | 00441246415097 |
| Newport animal centre | Monmouthshire (Wales) | No |  |
| North Cornwall Bird Rescue | Cornwall | No |  |
| Nuneaton & Warwickshire Wildlife sanctuary | Warwickshire | Yes | 00442476345243 |
| Oak and Furrow | Gloucestershire | Yes | 00441793751412 |
| Penwood Wildlife | Berkshire | Yes | 00441635253139 (sanctuary is no longer operating) |
| Putney animal hospital | Greater London | No |  |
| Quality of Life animal Sanctuary | Devon | Yes | 00441409253148 |
| Radcliffe Animal Trust | Nottinghamshire | No |  |
| Raven Haven | Berkshire | Yes | 00447831124464 |
| Riverside Animal Centre | Greater London | Yes | 00442086698877 |
| Roger's wildlife rescue | Sussex | No |  |
| Ryedale wildlife rehabilitation centre | Yorkshire | No |  |
| S&H Animal Rescue | Surrey | No |  |
| Saint Francis animal welfare | Hampshire | Yes | 00442380693282 |
| Saint Tiggywinkles | Buckinghamshire | Yes | 00441844292292 |
| Scarborough Wild Bird Rescue | Yorkshire | No |  |
| Scottish Island Sanctuary | Argyll and Bute (Scotland) | No |  |
| Seaham Owl sanctuary | Durham | No |  |
| Secret World wildlife rescue | Somerset | No |  |
| Selby Wildlife Rescue | Yorkshire | Yes | 00447803180720 |
| South Essex wildlife hospital | Essex | No |  |
| South Godstone animal centre | Surrey | No |  |
| South Yorkshire Animal Rescue | Yorkshire | Yes | 00441142349656 |
| Southridge animal centre | Hertfordshire | No |  |
| Surrey Bird Rescue Centre | Surrey | No |  |
| Swale Wildlife Rescue | Kent | No |  |
| Thanet Wildlife Rescue | Kent | Yes | 00441843844532 |
| The wildlife Haven | Yorkshire | Yes | 00441845526567 |
| The Berwick Swan and Wildlife Trust | Northumberland | Yes | 00441289302882 |
| The Bird Trust UK | West Midlands | No |  |
| The Fernwood Trust | Aberdeenshire (Scotland) | Yes | 00448445884477 |
| The Hawk Conservancy | Hampshire | No |  |
| The sanctuary wildlife care centre | Northumberland | No |  |
| The small pet rescue | Leicestershire | No |  |
| The wildlife haven | Yorkshire | No |  |
| The Wildlife Lodge | Kent | No |  |
| Thornberry animal sanctuary | Yorkshire | No |  |
| Three owls sanctuary & reserve | Lancashire | No |  |
| Tinkers Hill Swan & Bird of Prey | Pembrokeshire (Wales) | Yes | 00441834814397 |
| Tomar Owl Sanctuary | Kent | Yes | 00441233503758 |
| Trewitley Owl Trust | Northumberland | No |  |
| Turbary Woods Owl & Bird of Prey | Lancashire | Yes | 00441772323323 |
| Vale Wildlife Hospital & Rehabilitation Centre | Gloucestershire | Yes | 00441386882288 |
| Warwickshire Animal Sanctuary | Warwickshire | Yes | Sanctuary is no longer operating – no contact information could be retrieved |
| Warwickshire wildlife Sanctuary | Warwickshire | No |  |
| West Hatch Wildlife Centre | Somerset | Yes | 00443001230721 |
| Westport Wildlife Rescue | Derbyshire | Yes | 04401782811722 |
| Whitby Wildlife Sanctuary | Yorkshire | Yes | 00447342173724 |
| Wild Wings Birds of Prey Education & Rehabilitation Centre | Cheshire | Yes | 00447719717197 |
| Wildlife in need sanctuary | Derbyshire | No |  |
| Wildlife A&E | Sussex | No |  |
| Wildlife Aid | Surrey | No |  |
| Wildlife Rescue and Ambulance Service | Sussex | Yes | 00447815078234 |
| Leeds wildlife sanctuary | Yorkshire | No |  |
| Wildlife saving | Unknown | No |  |
| Wildlives | Essex | No |  |
| Willow Wildlife Rescue | Kent | Yes | 00447956472284 |
| Wiltshire wildlife Hospital | Wiltshire | No |  |
| Wing & a prayer, Wild bird and Owl Haven | Norfolk | No |  |
| Wings of freedom | Berkshire | No |  |
| Wolfwood Wildlife Rescue & Rehabilitation | Lancashire | Yes | 00447931220094 |
| Worthing and district animal rescue service | Sussex | No |  |
| Wythall animal sanctuary | West Midlands | No |  |
| Yorkshire Coast Wildlife Rescue | Yorkshire | Yes | Sanctuary is no longer operating – no contact information could be retrieved |
