# Supplementary material for: Investigating the impact of media on demand for wildlife: A case study of Harry Potter and the UK trade in owls
Source: PLoS One. 2017 Oct 4;12(10):e0182368. doi: 10.1371/journal.pone.0182368 (PMC5627891; doi:10.1371/journal.pone.0182368)
Supplement: S1 File — (DOCX) [file pone.0182368.s005.docx]

**S1 File. Raw data underlying this study**

This document provides the raw data underlying this study, when these are free to be released; and instructions for accessing identical information, when the raw data cannot be made public.

Harry Potter film ticket sales

Weekly summaries of the weekend box office gross incomes were collected from the archives of the British Film Institute (BFI, 2001-2011). From this dataset, the number of admissions per year was calculated by dividing the yearly total gross incomes by the average price of a ticket (The Cinema Exhibitors' Association, 2013).

| **Year** | **Number of cinema tickets sold for Harry Potter films in the UK** |
| --- | --- |
| 2001 | 9744341 |
| 2002 | 10199877 |
| 2003 | 738155 |
| 2004 | 8175802 |
| 2005 | 7139362 |
| 2006 | 257792 |
| 2007 | 6103118 |
| 2008 | 0 |
| 2009 | 5854805 |
| 2010 | 5822341 |
| 2011 | 7613284 |

Harry Potter book sales

Data concerning sales of Harry Potter’s books were obtained from Nielsen Book Services (<http://www.nielsenbookdata.co.uk/>). The raw data obtained belong to a third party and cannot be published here. We did not get special privileges to access this data and one could obtain identical information by contacting Nielsen Book Services. We requested yearly UK sales for all Harry Potter books, across all languages and versions from 1998 to 2013. The data were provided by Mr. Russell Bremner working in Nielsen’s Media Servicing and Analysis team on 12^th^ September 2014.

Harry Potter newspaper mentions

Data were collected from the LexisNexis database.

| **Year** | **Number of Harry Potter mentions in major UK newspapers** |
| --- | --- |
| 1997 | 11 |
| 1998 | 27 |
| 1999 | 191 |
| 2000 | 738 |
| 2001 | 1252 |
| 2002 | 1275 |
| 2003 | 143 |
| 2004 | 897 |
| 2005 | 1334 |
| 2006 | 973 |
| 2007 | 1583 |
| 2008 | 1114 |
| 2009 | 1306 |
| 2010 | 1341 |
| 2011 | 1911 |
| 2012 | 1663 |

Legal trade in owl

Data related to the magnitude of the UK trade in owls were obtained from three independent sources: the CITES database, and two main UK institutions supplying bird rings — the British Bird Council and the Independent Bird Register.

| **Year** | **Number of trade records for all owl genera in the CITES database** |
| --- | --- |
| 1990 | 1 |
| 1991 | 11 |
| 1992 | 0 |
| 1993 | 10 |
| 1994 | 8 |
| 1995 | 10 |
| 1996 | 0 |
| 1997 | 0 |
| 1998 | 2 |
| 1999 | 75 |
| 2000 | 57 |
| 2001 | 5 |
| 2002 | 16 |
| 2003 | 58 |
| 2004 | 40 |
| 2005 | 15 |
| 2006 | 0 |
| 2007 | 0 |
| 2008 | 0 |
| 2009 | 0 |
| 2010 | 0 |
| 2011 | 0 |

Information supplied by the British Bird Council cannot be released as they belong to a third party, although no special access privileges were given to us and identical data can be obtained by contacting the British Bird Council at [info@britishbirdcouncil.com](mailto:info@britishbirdcouncil.com). The monthly UK sale figures of rings size “U” and size “Z” from 1996 to 2012 were provided to us on 4^th^ February 2013.

For similar reasons, the raw data provided by the Independent Bird Register cannot be released here but can be obtained with no special access privileges by contacting the organisation at [enquiries@ibr.org.uk](mailto:enquiries@ibr.org.uk). Monthly data for Snowy owl rings that have been sold by the IBR in the UK from 1994 to 2012 were provided to us on 20th October 2012.

Abandonment of pet owls

To comply with the Data Protection Act (1998) and the University of Kent Code of Ethical Practice for Research, the answers to our survey from wildlife sanctuaries cannot be made public. This is owing to the fact that the participants’ consent for their responses to be made public was not requested. Consent was not originally sought since this survey was initially part of an undergraduate study which was not projected to be published.

A list of all 117 wildlife sanctuaries contacted between September 2012 and February 2013, with their respondent status and the contact information of those who took part in our survey can however be found in S1 Table, and the responses to our questionnaire can be made available upon request.
